# Differentiation of mixed biological traces in sexual assaults using DNA fragment analysis

**DOI:** 10.1080/13102818.2014.909171

**Published:** 2014-07-08

**Authors:** Аleksandar Apostolov

**Affiliations:** ^a^ Department of Forensic Medicine and Deontology, Faculty of Medicine, Medical University of Sofia, Sofia, Bulgaria

**Keywords:** material evidence, sexual abuse, mixed biological traces, DNA identification, Y chromosome

## Abstract

During the investigation of sexual abuse, it is not rare that mixed genetic material from two or more persons is detected. In such cases, successful profiling can be achieved using DNA fragment analysis, resulting in individual genetic profiles of offenders and their victims. This has led to an increase in the percentage of identified perpetrators of sexual offenses. The classic and modified genetic models used, allowed us to refine and implement appropriate extraction, polymerase chain reaction and electrophoretic procedures with individual assessment and approach to conducting research. Testing mixed biological traces using DNA fragment analysis appears to be the only opportunity for identifying perpetrators in gang rapes.

## Introduction

DNA fragment analysis in forensic work is increasingly necessary and meets the requirements of the justice bodies in the civil and criminal process. In the course of ongoing investigations of criminal offenses, and in particular, of sexual offenses, various items of evidence with biological traces on them are collected.[1] When only a small amount of biological material is available as material evidence, precise forensic tests using DNA fragment analysis have to be performed to detect the perpetrators of general crimes.[2–5] Another advantage of DNA fragment analysis is that it can be used for typing significantly degraded organic matter.

The combination of autosomal and sex-specific genetic markers and analysis of various types of tissues and secretions with available nuclei-containing cells (i.e. that contain DNA) is highly informative. This approach has a well-proven potential in the study of biological traces on material evidence.[6,7] In cases when the sexual offenses have been committed by more than one man, involving rape and fornication, and the investigation has found mixed biological traces, it is essential to perform DNA differentiation of the perpetrators of the act.[8,9]

Here we present and analyse data from our expert research and development work [10] performed using the method of DNA fragment analysis. We have performed successful DNA profiling of biological traces on material evidence and identifications of perpetrators of gang rapes. This has led to significant increase of the percentage of detected sexual offenses. The used models allowed us to apply some modified extraction, polymerase chain reaction (PCR) and electrophoretic procedures with individual assessment and approach to the study of mixed biological traces.[11–16]

## Materials and methods

In a total of 83 studied cases, we found 59 mixed biological traces of semen on the material evidence, and in four cases there was mixed material originating from more than two persons. There were four main types of mixed biological traces: saliva and semen in five cases; traces of semen mixed with vaginal discharges on vaginal smears and clothes in 37 cases; mixed traces of semen and blood on bed linen in 13 cases; and mixed traces of semen and rectal contents found on four of the surveyed sites. The traces of semen, saliva and mixed traces submitted for DNA profiling had originated more than three years before the profiling was performed.

The extraction of total DNA from mixed biological traces was carried out under an FBI report provided by LIFE TECHNOLOGIES (Debra Nickson, technical services; 29.01.97). Stain extraction buffer (0.01 mol/L Tris, 0.01 mol/L ethylenediaminetetraacetic acid (EDTA), 0.1 mol/L NaCl, 0.039 mol/L dithiothreitol, 2% sodium dodecyl sulphate) was used and Proteinase K (20 mg/mL) was added later. Organic (phenol) extraction (phenol: chloroform: isoamyl alcohol = 25:24:1) was carried out after an 18 h incubation at 56 °C. DNA precipitation was performed with absolute alcohol cooled to −20 °C. The extracted DNA was dissolved in 50 μL Tris-EDTA (TE) buffer and was stored at −20 °C.

The classic technique for differentiated extraction and separation of the sperm component from the vaginal contents (differential lysis) was applied for DNA extraction of mixed male/female biological samples.[17]

The blood samples taken from compared persons were processed for DNA extraction by the method of Roos and Loos [18] as described by Promega Corporation.[19] The extracted DNA was dissolved in TE buffer to a volume of 50 μL and was stored at −20°C.

We also did a comparative analysis of the PCR products obtained from biological traces on physical evidence (including mixed traces), using a new generation of Taq-polymerase (Platinium^®^ Taq DNA polymerase, Gibco BRL, licensed by Life Technologies, Inc., US patent N 5,338,671) that contains recombinant Taq DNA polymerase and an antibody inhibiting the effect of non-specific products from extracted samples.

We started amplification of the Short Tandem Repeats (STRs) markers in the tests of compared persons, using Ready.To.Go^®^ PCR Beads (Pharmacia Biotech): 1X Buffer, 1.5 mmol/L MgCl_2_, 0.2 mmol/L deoxynucleoside triphosphates, 1.5 U Taq-polymerase, 0.34 mg/mL bovine serum albumin, 0.4 pmol/μL Cy 5′ Primer A and Primer B (Pharmacia LKB), ddH_2_O and 10–90 ng of extracted DNA in a final volume of 12.5 μL. Standard control amplifications of DNA were performed with a known concentration of AmpFLSTR Positive Control DNA–human male 007 (0.10 ng/mL).

The concentration of DNA in the samples was measured using a Hoefer DyNAR Quant 200 (Amersham Pharmacia Biotech) fluorometer with a final concentration of 10–400 ng/mL, according to Waye et al.[20]

The resulting PCR products were analysed using 0.5 mm ReproGel™ (Amersham Biosciences) denaturing polyacrylamide (PAA) high-voltage electrophoresis with 8% acrylamide/bisacrylamide monomers and 1X Tris-borate-EDTA (TBE) buffer (0.1 mol/L Tris, 83 mmol/L Boric acid, 1 mmol/L EDTA), 1500 V, 60 mA, 30 W, 55°C. For uniform and faster gel solution polymerization, we applied the ReproSet™ (Amersham Pharmacia Biotech) ultraviolet laboratory tool for photopolymerization with 12 minutes fixed polymerization time.

We conducted the DNA fragment analysis on an automated laser sequencer ALFexpress™ DNA Sequencer (Pharmacia Biotech) using ultrathin (0.5 mm gel) PAA high-voltage electrophoresis on a standard thermocassette: 6% PAA gel, 0.65X Tris-borate-EDTA buffer (TBE: 1 mol/L Tris, 0.83 mmol/L boric acid, 10 mmol/L EDTA), 7 mol/L Urea ALF Grade, 1500 V, 60 mA, 25 W, 50 °C).

Control analyses were performed using: External standard sizer 50–500; internal standards (AMEL 106 bp and H16401-L16110 347 bp); and allele leaders for the corresponding STR markers, as described by Decorte.[21] The results were read with laser detection of fragments and computer analysis using Fragment Manager™ V1.2 software (Pharmacia Biotech).

## Results and discussion

The comparative studies of mixed biological traces, using the method of DNA fragment analysis, differentiated or identified 61 males for being the perpetrators in committed sexual offenses. We identified DNA profiled traces of biological origin left by two unidentified persons. Out of the total number of completed investigations, the expertise could not derive benchmarking genetic profiles in six cases.

**Figure 1.**
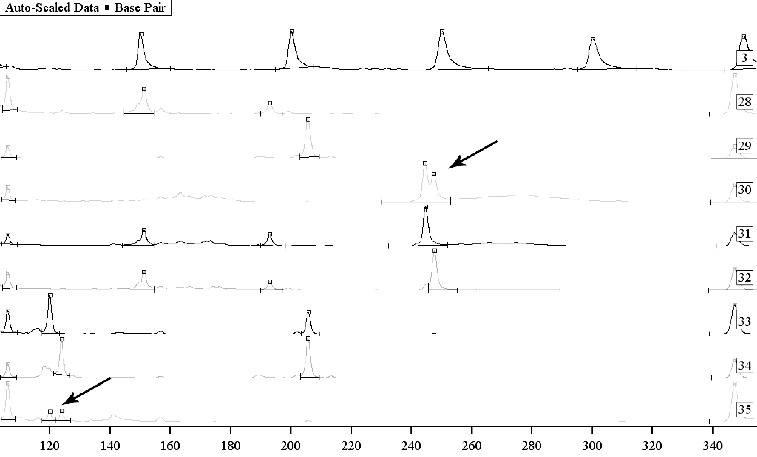
Mixed traces of semen from two men. Sample 3: Sizer 50–500. Samples 30, 31 and 32: a study on Y chromosome marker DYS392 with sequencing – semen on sports clothes and two suspects. Samples 33 and 34: a study on Y chromosome markers DYS393 and DYS390 with sequencing – two suspects, and sample 35: mixed semen from two men on physical evidence. Samples 30 and 35: mixed biological material where differentiation of the DNA profiles of the two persons was possible.

Our results showed that Platinium^®^ Taq DNA polymerase is well balanced, which allowed us to obtain high-quality PCR products for all the living persons tested. The Ready-To-Go^®^ beads were found appropriate to the study of clean (not mixed) traces but were less reliable for amplification of DNA from contaminated or mixed traces of unknown proportion. The more successful approach in such cases – as previously noted [22] – appeared to be the Platinium^®^ Taq DNA polymerase, which showed good qualities and inhibited non-specific products in approximately 85% of the samples with isolated DNA. The advantage was reflected in the visualization of smaller and lower extra peaks with good vertical and horizontal balance of the allele peaks. The results demonstrate the possibility for differentiation of the two men perpetrators in a gang rape (Figure 1). The unsuccessful amplifications in six samples were probably due to insufficient quantity or fragmentation and major DNA degradation.

**Figure 2.**
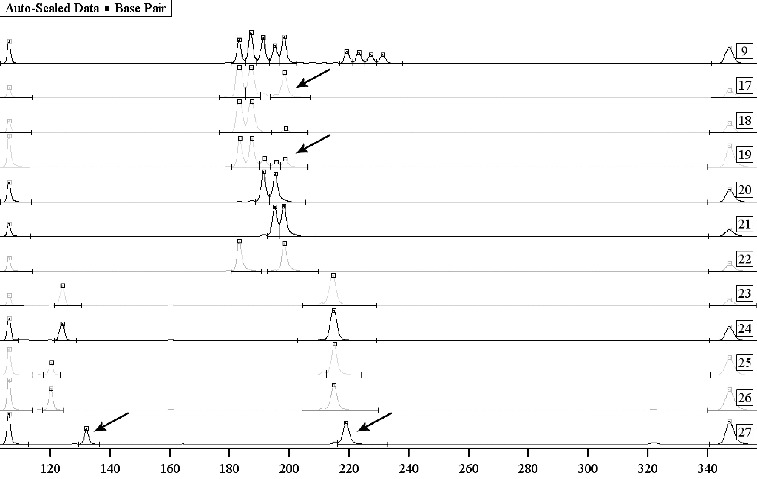
Mixed traces of semen – Y-chromosome exclusion of complicity. Sample 9: allele witness using autosomal markers TH01 and FES. Samples 17**–**22: testing using autosomal marker TH01 – vaginal smear, briefs, the victim and three suspects. Samples 17 and 19: mixed biological material with no potential for the differentiation of the DNA profiles. Samples 23**–**27: testing of biological material from four suspects, using Y chromosome markers DYS393 and DYS390, with a potential for the exclusion of a suspect as the perpetrator – sample 27.

Using our technique for total DNA extraction from mixed biological traces, we obtained Y-chromosome typing of more than one perpetrator of sexual crime. We used a set of 10 Y-chromosome markers. Y-chromosome profiling of traces of semen from two men in post-coital samples resulted in their differentiation in the composite sample. The male Y-chromosome marker identification performed in cases of gang rapes with more than two perpetrators, showed a definite result in the exclusion of a person as an accomplice to the crime under investigation (Figure 2).

The tests to detect the DNA profile on the basis of Y-chromosome markers, carried out on mixed samples, showed the extremely good prospects of this approach, making it possible to obtain clean amplification products, regardless of the present target DNA from the victim. All this proves the invaluable role of gender-differentiated STR markers when used for detection of sex offenders.

However, our results from the analysis of electrophoretically separated PCR products showed that, in spite of the standardized implementation of all steps of separation/extraction in traces of post-coital origin, the separation of biological material is incomplete in most cases. There were mixed DNA profiles of the victim and the perpetrator in which it was difficult to interpret the results due to overlapping of bands of identical alleles. The problem is of particular importance in biological material left by more than two perpetrators (Figure 2). The basis for such an effect is the presence of mucosal epithelial cells from the perpetrator in mixed samples, especially in samples of vaginal smears.

In total extraction of mixed samples, the existing quantitative ratios of the mixed biological material give different concentration of extracted DNA. This can be demonstrated with peaks of different sizes reported in the laser detection of the corresponding loci of allele distribution based on a given marker. However, there is a chance for overlapping of bands when the PCR products have the same electrophoretic characteristics, which also limits the identification of DNA profiles of individuals who have left the traces.[23]

Another approach for successful profiling in rape committed by one perpetrator is based on autosomal genetic markers. It may allow the differentiation of the male from the female genetic material in mixed traces. For example, we reported positive results with the possibility for differentiation of the DNA profiles, using autosomal genetic markers, from mixed organic material from one perpetrator and the victim.

**Figure 3.**
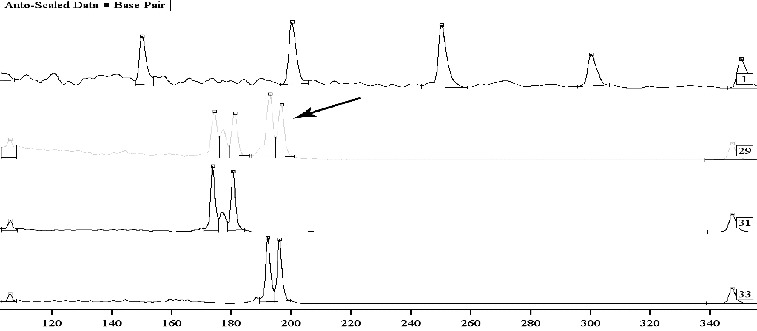
Mixed traces – semen from a man, in the vaginal contents. Sample 1: Sizer 50–500. Samples 29, 31 and 33: tested using an autosomal marker CYAR04 with sequence – vaginal smear, suspect and victim. Sample 29: mixed biological material with the potential for differentiation of the DNA profiles of the two parties.

Successful expertise in cases of investigation of traces of semen, saliva and mixed traces can be obtained using the method of total DNA extraction from a trace in order to reduce the loss of DNA material when minimum quantities are usually available and when it is impossible to repeat the study. In some of these cases, DNA was successfully identified using the method for total DNA extraction from mixed traces containing sperm (Figure 3).

The knowledge of the possible locations of biological traces deposited on the objects ensures the correct approach for finding the minimum quantity of biological material. In our experience, this holds true even in cases of searching for ‘blind’ micro traces.[24]

## Conclusions

Our results from the analysis of mixed biological traces in sexual offenses demonstrated how Y-chromosome profiling of traces of semen from two or more men in post-coital samples in cases of gang rapes allows their identification in a composite sample. Successful DNA profiling based on Y-chromosome markers may also give definite results in the exclusion of a person as an accomplice in gang rapes with more than two perpetrators. The set of Y-chromosome markers used by us allowed the differentiation of male genetic profiles from mixed biological traces, i.e. the so-called ‘male identification’. The procedures used gave successful DNA profiling in examination of traces taken three or more years before the analyses. All this illustrates the invaluable role of gender-differentiated STR markers when used for detection of sex offenders.
